# Experiences of family caregivers of people with intellectual disabilities from rural areas in southeastern Iran: a qualitative study

**DOI:** 10.1186/s12888-023-05077-0

**Published:** 2023-08-22

**Authors:** Raziyeh Sadat Bahador, Jamileh Farokhzadian, Farshid Rafiee Sarbijan Nasab, Mohsen Abbasi

**Affiliations:** 1 Department of Nursing, School of Nursing and Midwifery, Jiroft University of Medical Sciences, Jiroft, Iran; 2 Nursing Research Center, Kerman University of Medical Sciences, Kerman, Iran

**Keywords:** Achievements of care, Family caregivers, Intellectual disability, People with intellectual disabilities, Qualitative study

## Abstract

**Background:**

Caring for people with intellectual disabilities can be a challenging task, but it can also bring about positive experiences for family caregivers. The present study aimed to explore these positive experiences and shed light on the ways in which family caregivers of people with intellectual disabilities can find meaning and fulfillment in their roles.

**Methods:**

This qualitative study used conventional content analysis to explore the positive experiences of family caregivers of people with intellectual disabilities. Sixteen family caregivers from rural areas in southeastern Iran were purposively selected to participate in the study. Semi-structured in-depth interviews were conducted to collect data. The interviews were audio-recorded and transcribed verbatim. The data were analyzed using the method proposed by Graneheim and Lundman.

**Results:**

The data analysis resulted in the emergence of a single theme, which was achievements of providing care for people with intellectual disabilities. This theme was comprised of four main categories: a new outlook on life, family caregivers’ peace of mind, strengthening of family ties, and improvement of social status. The provision of care for people with intellectual disabilities created positive changes, affecting family functioning and care provision for people with intellectual disabilities.

**Conclusion:**

It is recommended that families of people with intellectual disabilities focus on these positive experiences and share them with other families who may be struggling with a recent diagnosis of intellectual disability in their child.

## Background

Intellectual disabilities are characterized by below-average intelligence and insufficient skills in daily life, including routine tasks that a person typically performs at a particular age [1]. This disorder typically manifests before the age of 18 and affects between one and three% of people in the general population, with varying degrees of severity [2, 3].

The International Classification of Functioning Disability and Health for Children and Youth (ICF-CY) was developed to provide a comprehensive framework for understanding the health and functioning of children and youth with disabilities [4]. At the heart of the ICF concept of health and disability is the idea that disability is a multidimensional and universal phenomenon that exists on a continuum with health. Human functioning is viewed as a continuum of health states, and every human being exhibits one or varying degrees of functioning across different domains, including the body, person, and society [5]. The ICF emphasizes that disability is not solely as a problem that resides in the individual but is a health experience that occurs within a particular context. Disability and functioning are consequences of interactions between health conditions (diseases, disorders, and injuries) and contextual factors. The bio-psychosocial model embedded in the ICF allows for a broader perspective on disability, enabling medical, individual, social, and environmental influences on functioning and disability to be examined [5–7]. Therefore, it is crucial to examine the responses and reactions of families of people with disabilities as they are one of the most significant factors influencing their growth and performance.

In Iran, the well-being organization serves as a support system for families with disabled children by providing financial, medical, rehabilitative, and educational services, counseling, and insurance, thereby playing a crucial role in promoting the independence and empowerment of people with disabilities [8–10]. However, in rural and remote areas, families may face challenges in accessing support services beyond financial assistance and insurance [11].

Several studies have been conducted in Iran that highlight the negative consequences of caring for people with intellectual disabilities. These studies reveal that families of people with intellectual disabilities experience high levels of mental pressure, leading to caregiving challenges and particularly stressful experiences [12–15].Recent studies have also highlighted the positive effects that providing care for people with intellectual disabilities can have on family caregivers. For example, some caregivers reported experiencing a sense of enjoyment and fulfillment from providing care, which encouraged them to continue their caregiving responsibilities [16, 17].

The concept of adaptation and acceptance is crucial to achieving positive experiences. Adaptation to disability is an ongoing process in which individuals gradually move towards a more favorable and harmonious relationship with their environment [16]. While adaptation emphasizes interactions with the surrounding world, acceptance focuses on an individual’s self-concept [17]. Both processes involve changing values and are necessary for individuals who willingly adapt to life changes [16, 17]. A review of the literature suggests that family caregiving for people with intellectual disabilities can be a positive experience when coping strategies and flexibility are used [17, 19]. By using effective coping strategies and adapting to the concept of disability, caregivers can move from the challenging and distressing stage of adjusting to disability to the stage of effectively fulfilling their caregiving roles [17].

Caregivers have reported feeling satisfied and experiencing a sense of vitality when caring for their loved ones, and have identified positive achievements such as personal growth, learning new skills, and becoming more determined to face challenges [20, 21]. Other studies have highlighted the positive impact that caregiving can have on family relationships, including strengthening family bonds and promoting a sense of social responsibility [22, 23]. Despite these positive experiences, much of the research on caregiving for people with intellectual disabilities has focused on its negative aspects. However, it is important to recognize the multidimensional nature of caregiving and to consider both its positive and negative aspects [18, 19].

Given the limited availability of support services for rural families in southeastern Iran and the additional challenges they face in providing care compared to families in urban areas, the present study was conducted to explore the experiences of rural families regarding the positive consequences of caring for people with intellectual disabilities.

## Methods

### Study design and setting

This qualitative study was conducted using a conventional content analysis method from September to December 2022. This systematic method was applied to describe a phenomenon deeply, release experiences, and determine its patterns and communication processes [20].

The study was conducted in the villages of southeastern Iran, an area known for its poverty and deprivation, particularly in rural regions. These villages were at least 50 and at most 100 km away from the city center, making it challenging for residents to access urban facilities.

### Participants

The participants were selected purposefully, with an emphasis on maximum variation sampling. To obtain a more comprehensive understanding of the experiences, we considered diversity in socio-economic status, level of education, severity of the disability of people with disabilities in the family, and the type of relationship between the caregiver and people with disabilities.

The inclusion criteria for participants are as follows: at least one year of experience in caring for a disabled person, favorable mental health, and a desire to share their experiences. Additionally, participants should not have any physical disabilities that would prevent them from engaging in self-care and should not have any known mental disabilities.

The study purpose was first explained to eligible participants. For those caregivers who expressed hesitation in participating, a follow-up appointment was scheduled to provide more information and answer any questions they may have had. Participants were reassured that any information regarding the individual with intellectual disabilities would be kept confidential by the research team. Additionally, all families were appreciated for their involvement by receiving a gift for people with disabilities. Data collection continued until data saturation was achieved, which occurred after the 14th interview. However, two additional interviews were conducted to increase for more clarification. Table 1 provides demographic information about the participants.

**Table 1:** Participants’ demographic characteristics

Characteristics	Category (n)
Caregiver’s relationship with the person with an intellectual disability	Mother (n = 6) Father (n = 3) Sister (n = 3) Brother (n = 3) Grandmother (n = 1)
Caregiver’s age	Less than 20 (n = 1) 20–30 (n = 3) 31–40 (n = 4) 41–50 ( n = 4) 51–60 ( n = 3) More than 60 (n = 1)
Caregiver’s education level	Uneducated (n = 3) Diploma (n = 8) Bachelor’s (n = 5)
Family income	Poor (n = 4) Moderate (n = 9) Good (n = 3)
Disability degree of the person with an intellectual disability	Mild (teachable) (n = 1) Moderate (highly teachable) (n = 11) Severe (less teachable) (n = 4)
Age of the person with an intellectual disability	1–5 (n = 4) 6–10 (n = 5) 11–15 (n = 3) 16–20 (n = 3) 21–23 (n = 1)
Sex of the person with an intellectual disability	Girl (n = 8) Boy (n = 8)

### Data collection

Data were collected through semi-interviews with 16 participants. Proper relationships were established between the identified categories. The first author conducted interviews, but all researchers reviewed and studied the interviews to identify their strengths and weaknesses. The subsequent interviews were adjusted based on this review process. To ensure a comprehensive exploration of the positive experiences of family caregivers, additional questions were added to the interview protocol that specifically addressed this topic.

The interview guide questions included:

1) Can you describe your experiences of caring for people with disability?

2) According to your own experiences, what actions and factors encourage you to be more caring for people with disabilities?

3) Would you have had these experiences without the presence of a person with intellectual disabilities in your life?

4) In your experience, what are the positive consequences of caring for people with intellectual disabilities?

Follow-up questions were based on the information provided by the participants to seek clarification and deeper understanding such as “What do you mean?” “Can you explain more?” and “Can you give an example?”

Each interview took 45–90 min. At the end of each interview, the interviewer gave her phone number to the participants in case they needed to provide additional information or had any question. The interviews were conducted in places preferred by the participants (private homes, parks, nursing schools, etc.).

### Analysis

We collected and analyzed data simultaneously and used MAX Qualitative Data Analysis 20 to organize and compare data. The transcript of each interview was reviewed several times and the Graneheim and Lundman’s content analysis process was used to transcribe the interviews, obtain a general perception of their content and get immersed in the data. Meaning units were condensed, primary codes were extracted, similar primary codes were classified under the same subcategories, and similar codes were classified under more comprehensive categories. Latent and manifest concepts were extracted, and final themes were formulated [21]. Table 2 showed subcategories based on their conceptual similarity, abstract categories (main categories) and theme.

**Table 2:** An example of formation of a category

Category	Subcategories	Examples of codes	Examples of meaning units	Examples of quotations
A new outlook on life	Paying in-depth attention to the nature of life	Family members obtained deep insight, believed in divine test, thanked God after adversities, and placed a high value on health and eternal life.	Family members changed their superficial views on life issues after facing difficulties, believed that God tested his servants through their resistance against adversities, thanked God when facing adversities and attributed them to God’s wisdom, placed a high value on health and a trivial value on material life due to the importance of the eternal world.	I think hardships change a person’s perspective. I used to see everything superficially, for example, I thought that people who always faced hardships in their lives were miserable and unlucky, but now I know that God tests his servants with suffering; I used to complain to God a lot, but now when I see a difficulty, I just thank God for his wisdom. When I see my granddaughter’s mental and physical condition, I understand that health is the most valuable property; the only thing that is important to me is health and peace. The material belongings are not valuable to me because this material life is limited and ends in the blink of an eye, while eternal and true life is immortal.
	Restarting life after experiencing difficult events	Caregivers became independent to solve problems, became experienced and had fighting spirit, learnt and memorized lessons from past difficulties.	Caregivers solved their problems independently, stood on their own feet, became stronger than the past, underestimated new problems, and memorized problems related to disability	Now, I stand on my own feet and can handle many of my problems alone, whereas I was weak in the past and I could become tired of the smallest problems; we suffered a lot during these years. When a problem occurs in my life, I quickly remember the day when I had just found out that my sister was intellectually disabled; I think to myself that this problem is not as big as my sister’s problem and I have to handle it. Therefore, my problems become trivial and unimportant; I do not forget those days.

The second and fifth authors extracted, reviewed, and approved codes and categories. The researchers continuously analyzed and compared the data to reduce the initially extracted codes. Lincoln and Guba’s criteria (credibility, dependability, confirmability, and transferability) were used to ensure the data trustworthiness [22]. For credibility, we asked the participants to review and confirm the codes and categories extracted from the interviews and revise the contents (member check). To enhance the confirmability of the findings, the second, third, and fifth authors (peer check), as well as a faculty member who was not a member of the research team (faculty check), reviewed and confirmed all texts of the interviews, codes, and categories. To ensure the dependability of the results, we recorded all stages of the study and used maximum variation sampling to select the participants, which enhanced the transferability of the study findings.

## Results

We conducted interviews with 16 family caregivers of people with intellectual disabilities, who had positive caring consequences (six mothers, three fathers, three sisters, three brothers, and one grandmother of people with an intellectual disability). Eight families had moderate incomes, five had poor incomes, and three had good incomes. People with intellectual disabilities included eight boys and eight girls aged 1–23 years. Of the 16 people, 11 had a moderate disability, four had severe disabilities, and one had a mild disability (Table 1).

Our participants reported experiencing negative consequences of caregiving such as high psychological pressure, stress, anxiety, depression, and other negative symptoms. However, the overall goal of our study was to explore the positive consequences of caregiving, which demonstrated the families’ acceptance and adaptation to caring for a family member with disability.

The theme emerged was the achievements of caring for people with intellectual disabilities that included four main categories: a new outlook on life, family caregivers’ peace of mind, strengthening of family ties, and improvement of social status (Table 3).

**Table 3:** Theme, categories, and subcategories extracted from the data

Theme	Categories	Subcategories
Achievements of caring for people with intellectual disabilities	A new outlook on life	-Restarting life after experiencing difficult events -Paying in-depth attention to the nature of life
	Family caregivers’ peace of mind	-Satisfaction with rehabilitation of the people with intellectual disabilities -The family members’ peace of mind due to providing proper care to the people with intellectual disabilities -Adaptation and acceptance of the people with intellectual disabilities
	Strengthening of family ties	-Close family relationships -A sense of security, support, belonging, responsibility in the people with intellectual disabilities -Institutionalization of positive traits in family members
	Improvement of the social status	-Modeling and social approval of the family members among the people around them -Improvement of family members’ social relations, social support of the people with intellectual disabilities

### A new outlook on life

This category represented a new intellectual insight the family caregivers acquired when providing care for a person with severe intellectual disability. This category included the subcategories of paying in-depth attention to the nature of life and restarting life after experiencing difficult events.

#### Paying in-depth attention to the nature of life

Participants believed that unfortunate events changed their insights and attitudes towards the nature of life. As a result, they expressed a desire to change certain behaviors such as complaining and ingratitude.

*“I think hardships change a person’s perspective. I used to see everything superficially, for example, I viewed people who faced ongoing struggles as being unlucky and miserable, but I now realize that difficult experiences can serve as a test of faith and an opportunity for personal growth. In the past, I would often complain to God when faced with challenges, but I have learned to approach difficult situations with gratitude and trust in God’s wisdom.” (6)*

Caring for people with intellectual disabilities caused family caregivers to shift their focus away from material possessions and towards the spiritual aspects of life. Caregivers reported thinking more about the eternal world and the teachings of divine religions and prophets.

*“When I see my granddaughter’s mental and physical condition, I am reminded of the value of health as the most precious possession. Material belongings no longer hold the same importance to me, as I recognize that this material life is finite, while the eternal and true life is everlasting.” (8)*

#### Restarting life after experiencing difficult events

Based on the perspectives shared by study participants, facing difficulties, failures, and other challenges helped them to develop resilience and strength.

*“I have become much more self-sufficient and capable of handling my own problems than I was in the past. Even the smallest problems would leave me feeling weak and exhausted.” (7)*

Participants in the study reported that they gained valuable insights and lessons from their difficult experiences, and that they tried to keep these lessons in mind to help them cope with future challenges.

*“When I encounter a problem in my life, I am reminded of the time when I first learned that my sister was intellectually disabled. This memory helps me to put my own problems into perspective and recognize that they may not be as significant as other challenges I have faced. As a result, my problems seem less daunting and I feel better equipped to handle them.” (5)*

### Family caregivers’ peace of mind

According to the findings, taking care of people with intellectual disabilities made family caregivers satisfied and calm. This category was mostly true about mothers and included subcategories of satisfaction with the rehabilitation of the people with intellectual disabilities, family caregivers’ peace due to providing proper care for the people with intellectual disabilities, and adaptation and acceptance of the people with intellectual disabilities.

#### Satisfaction with the rehabilitation of the people with intellectual disabilities

The study participants reported that care, prevention, and rehabilitation caused the people with intellectual disabilities to progress in different aspects of life; caregivers were pleased because he/she could meet his/her daily needs without their assistances.

*“I am very happy to see that he does most of his personal work. Previously, we had to assist him with basic tasks such as eating, toileting, and bathing. I am very happy and feel comfortable that he can handle most of his daily needs in my absence.” (15)*

Some participants were satisfied with their caregiving because they could search talent and train the people with intellectual disabilities in the fields they were interested.

*“We became aware that my brother had a strong interest in both carpet weaving and agriculture, and we decided to support him in pursuing these passions. Over time, he has made significant progress in both areas and has been able to establish a reliable source of income for himself and his family.” (16)*

#### Family caregivers’ peace due to providing proper care for the people with intellectual disabilities

Some families felt a heavy responsibility for the people with intellectual disabilities and they experienced a deep inner peace by providing proper care for them.

*“As a mother, I find a great sense of calm and satisfaction when I am able to fulfill my responsibilities towards my daughter. This peace of mind is a valuable and essential aspect of my life, and I feel grateful to have been given the opportunity to be a mother.” (14)*

On the contrary, they felt guilty when they failed to undertake their responsibilities for the people with intellectual disabilities, a feeling that was versus a sense of peace that resulted in inner satisfaction in the participants.

*“I feel excellent when I undertake my responsibilities for him well. However, I also feel guilty when I find myself getting frustrated or angry with him such as when he touches my belongings. Although I have experienced both of these feelings, I strive to avoid doing anything that may cause me to feel guilty later.” (10)*

#### Adaptation and acceptance of the people with intellectual disabilities

Acceptance of disability is a positive aspect of providing care for a child with intellectual disability among family caregivers. The study participants acknowledged that accepting God’s will and desire made them adapt to the condition of the people with intellectual disabilities. This acceptance allowed them to move beyond the challenges and difficulties of caring for a person with a disability.

*“When I first learned about his disability, I was in disbelief and felt overwhelmed by guilt and self-blame. The more I thought about it, the more confused and distressed I became. However, over time, I have come to accept that it is part of God’s plan, and this realization has brought me a sense of calm and peace.” (1)*

According to some participants, if one family member accepts people with intellectual disabilities, all family members will accept and adapt to the condition.

*“When my mother would cry because of my brother’s disability, both my brother and I would be deeply affected and experience psychological distress. However, with the help of a counselor, my mother was able to better manage her feelings and find a way to cope with the challenges of caring for a child with a disability. This support was beneficial not only for my mother but for our entire family.” (3)*

### Strengthening of family ties

This category suggested that caring for people with intellectual disabilities strengthened family ties and relationships. This category included the subcategories of close family relationships, a sense of security, support, belonging, and responsibility for the people with intellectual disabilities, institutionalization of positive traits in family members.

#### Close family relationships

Caring for people with intellectual disabilities had a positive impact on family dynamics, leading to increased quality time spent together and a more intimate family atmosphere. These improved relationships, in turn, had a beneficial effect on the people with intellectual disabilities, improving their physical, mental, psychological, and emotional well-being.

*“I was employed, but I became a work-from-home employee due to my daughter’s condition. I feel closer and friendlier with my family members as I spend more times with them. In particular, my daughter and I have grown closer due to the increased time we spend together.” (7)*

Caring for people with intellectual disabilities can lead to increased dependence and attachment among family members. Living with a person with a disability often creates strong emotional bonds between family members and the individual, and in some cases, they may find it difficult to imagine life without them.

*“We will miss her when she is not at home; we feel her absence at home. This was particularly evident when my sister spent two weeks in a center for child and family well-being. During this time, the house was unusually quiet and silent, and we all missed her presence immensely.” (4)*

#### A sense of security, support, belonging, and responsibility for the people with intellectual disabilities

This subcategory was another achievement of family caregivers that played an important role in creating strong family ties.

*“Despite his mental health condition, my brother is able to communicate effectively with us and to express his trust in our family. When he is upset by someone outside of our home, he turns to us for support and protection.” (13)*

Some of the participants reported that involvement of the people with intellectual disabilities in familial tasks increased their self-awareness, sense of responsibility and belonging.

*“One of the positive aspects of having a supportive family is the impact it can have on the mental and emotional well-being of people with disabilities. For example, my daughter often tries to bring joy and happiness to our home by surprising me with her kindness and thoughtfulness.” (2)*

#### Institutionalization of positive traits in family members

This subcategory strengthened family relationships that was one of the important achievements of caring for the people with intellectual disabilities.

*“When I look at similar families, I am proud to say that my own family is truly exemplary in caring for a person with disability. While a center for child well-being may have been able to provide protection and support for my sister, my parents chose to keep her within our family, despite the challenges that came with this decision.” (13)*

Parents’ sacrifice was one of the factors affecting the emotional relationships of other family members.

*“The family environment can have a profound impact on a person’s behavior, and I have learned this firsthand through my experiences with my own family. Seeing how my parents love and support my disabled sister has had a significant impact on the way I approach my own relationships and interactions.” (4)*

### Improvement of social status

This category suggested the strengthening of the social relations and status of the people with intellectual disabilities and the family at the community and included the subcategories of modelling and approving family members among the people around them, improving the social relations of family members, and supporting the people with intellectual disabilities to engage in the society.

#### Modeling and approving family members among the people around them

The family members’ success in supporting, providing care for, and empowering people with intellectual disabilities led to their approval and modeling among similar families. Some families who had recently delivered a child with disability sought help from families who were more successful in this regard and referred to them as good role models.

*“Since we have communicated with similar families, I can see its impact in our life more, which is why I always try to model families that have achieved greater success.” (11)*

Families who served as role models attempted to offer guidance to families facing similar circumstances.

*“As a father, I have always tried to undertake my responsibilities for my child and give advice to families with similar conditions; I try to behave correctly so as not to be a bad role model.” (12)*

#### Improving social relations of family members

Although family members had a sense of inferiority, low self-esteem, and social isolation when receiving negative reactions from the society, they resisted against these reactions using appropriate defense mechanisms.

*“I used to hide my sister from my friends because I thought that they would cut their relationship with me. This act of hiding my sister had an adverse effect on my self-esteem, so I made the decision to open up about her to my friends. Now, I feel my self-confidence and relationship have improved.” (13)*

#### Supporting the people with intellectual disabilities to engage in the society

People with intellectual disabilities receive greater attention from caregivers because of the stigma associated with their condition. It is crucial for families to provide better support for people with disabilities in society, so that the negative perception of disability transforms into admiration within the community.

*“At first, I felt very unhappy when people in my community looked at my sister with pity. To counter this negative perception, I supported my sister in pursuing her interests. As a result of her achievements, my sister is now widely recognized in our village as a capable individual. In fact, she was even interviewed on television.” (4)*

Some of the study participants believed that some people in the society stigmatized people with disability and made them isolated, but family members could prevent the effect of social stigma on people with intellectual disabilities.

*“I recall an incident where I observed from a distance as my disabled brother was harassed and ridiculed by some people on the street. This experience left him feeling fearful of going outside and socially isolated. Despite our family’s concerted efforts to boost his self-confidence, such unpleasant encounters persisted. We tried to make him less affected.” (10)*

## Discussion

The study results suggested that family caregivers of the people with intellectual disabilities experienced positive consequences, which included the four main categories of a new outlook on life, family caregivers’ peace of mind, strengthening of family ties, and improvement of social status. Although the majority of studies conducted in Iran have focused on the negative consequences of caring for patients with disabilities such as psychological pressure and stressful events for families with children with mental retardation, they have also recommended support services to reduce the burden of care and foster acceptance of disability [12–14, 23, 24]. However, Naghavi et al. (2019) showed that while negative consequences of caregiving existed, coping strategies and adaptation could help caregivers move from the difficult stage of accepting disability to the stage of adaptation and positive caring roles [25]. Based on the participants’ experiences, the sufferings that families endure in taking care of the patient have led to positive consequences and changes in their lives.

Inan Budak et al. (2018) indicated that although all mothers of children with disabilities were worried about their children’s future and experienced significant changes in their social and family relationships, they had a positive perception of having a child with intellectual disability. Some of these mothers had no problem when caring for their children [26]. Kimura et al. (2013) found that mothers shifted their focus from caring for their disabled children to creating a society where their children could thrive [27]. These authors supported the present study results, which found that caregivers underwent a transformation in their outlook on life. Other studies suggested the formation of a new identity [28], a shift in priorities [27, 28], the promotion of spirituality [29–31], and efforts to overcome challenges [32, 33]. These factors were associated with the category of a new outlook on life in the present study, with spiritual beliefs playing a significant role in changing the perspective of caregivers. The results of the studies indicated that most of caregivers evaluated their situation through a religious lens and perceived themselves as fortunate servants of God or viewed their children as a blessing from God. Strengthening their spiritual beliefs enabled caregivers to view the presence of disabled children in a positive light [29–31, 34]. The deep religious beliefs and Islamic culture prevalent in the region provided a justification for strong beliefs in the afterlife. Family members of people with severe disabilities often turned to religious beliefs, possibly as a coping mechanism for their challenging living conditions and sense of helplessness.

The study participants believed that caregivers’ peace of mind was one of the positive achievements of caring for people with intellectual disabilities. Adithyan et al. (2017) demonstrated that the progress and appropriate behavior of people with intellectual disabilities had positive consequences that reduced family members’ stress and mental health problems [32]. Some studies reported that caring for people with intellectual disabilities created peace of mind in the family caregivers [26, 35–37], which confirmed the participants’ experiences in the present study. Peace of mind was a valuable experience that helped them cope with stressful challenges [32, 35]. Various studies supported our results and reported adaptability [32, 35], satisfaction and vitality [31, 38] and high motivation [29, 30, 32], which were associated with caregivers’ peace of mind. According to some studies, parents believed that they had been entrusted by taking care of a child with intellectual disability, so they had no problem in accepting and adapting to the child’s condition and had a high willingness to care for and train the child [30, 31]. The participants in the present study believed that accepting God’s will lead them to better adapt and accept the people with intellectual disabilities and tolerate difficulties better.

Strengthening of family ties was another result of the present study. According to Budak Inan, some mothers of children with intellectual disabilities acknowledged that their husbands increased their mutual support and became more dependent on each other [26]. Other studies also identified a child with intellectual disability as a factor strengthening family ties [32, 39]. Sato et al. (2015) mentioned that increasing family members’ teamwork and learning to manage the challenges of caring for these children were rewards for taking care of disabled children [40]; this result was consistent with the present study results.

Improvement of social status of the people with intellectual disabilities and family members was another result of the present study. Adithyan et al. (2017) introduced social responsibility as an important positive consequences in the care of people with intellectual and physical disabilities, so parents could improve their social contributions and gradually cope with social challenges [32]. Some studies suggested that blame and pity limited parents’ social relationships rather than the presence of disabled children, meaning that parents reduced their relationships with families, who behaved their children improperly, while their social approval and modeling among the people around them improved [26, 30, 35]. Social stigma of disability in the present study caused family caregivers to pay attention, support and care for the people with intellectual disabilities.

Research conducted worldwide indicates that societies in both Eastern and Western regions still require greater awareness to mitigate negative experiences and enhance positive experiences for parents of people with intellectual disabilities. It is still necessary to optimize the performance of specialized centers and support people with intellectual disabilities and their families [41].

Health professionals should help family caregivers better respond to their challenging roles and correct their insights. The present study reported a set of caregiving benefits, which can be used to design appropriate interventions for families and their disabled children. Authorities should design interventions using domestic resources and focus on family abilities such as personal characteristics, religious and spiritual factors, and social and family relationships to facilitate the care process.

### Limitations

The present study was performed in southeastern Iran, so more studies are necessary to generalize it to other cultural groups. As families took care of patients at home, we were unable to observe all behaviors of family members at the desired times. The researcher tried to control this limitation by long engaging with the family members and gaining their trust, so that they could express their behaviors freely.

It is important to note that this study is part of a larger research project that examines both the positive and negative experiences of family caregivers. Due to the word limit of this paper and the focus of the present study, only the positive consequences have been presented. The research team is going to report on the negative consequences of caregiving in a future study.

## Conclusions

The study results showed that living with and providing care for people with intellectual disabilities could have positive consequences for different family members and even for the disabled person. The challenges of life and family experiences in caring for people with disabilities have led to positive changes in family perceptions. As a result, examining the valuable experiences of the family members in caring for people with intellectual disabilities and their strengths can help researchers design and develop family interventions for better care of people with intellectual disabilities.

## Data Availability

The datasets used and/or analyzed during the current study available from the corresponding author on reasonable request.
